# Delving into the Aftermath of a Disease-Associated Near-Extinction Event: A Five-Year Study of a Serpentovirus (Nidovirus) in a Critically Endangered Turtle Population

**DOI:** 10.3390/v16040653

**Published:** 2024-04-22

**Authors:** Kate Parrish, Peter Kirkland, Paul Horwood, Bruce Chessman, Shane Ruming, Gerry McGilvray, Karrie Rose, Jane Hall, Lee Skerratt

**Affiliations:** 1 Virology Laboratory, Elizabeth Macarthur Agriculture Institute, Department of Primary Industries, Menangle, NSW 2568, Australia; 2 College of Public Health, Medical and Veterinary Sciences, James Cook University, Townsville, QLD 4810, Australia; 3 Chessman Ecology, Pymble, NSW 2073, Australia; 4 Biodiversity Conservation and Science, New South Wales Department of Climate Change, Energy, the Environment and Water, Coffs Harbour, NSW 2450, Australia; 5 Australian Registry of Wildlife Health, Taronga Conservation Society Australia, Mosman, NSW 2088, Australia; 6 Melbourne Veterinary School, Faculty of Science, University of Melbourne, Werribee, VIC 3030, Australia

**Keywords:** turtle, reptile, wild, serpentovirus, nidovirus, epidemiology

## Abstract

Bellinger River virus (BRV) is a serpentovirus (nidovirus) that was likely responsible for the catastrophic mortality of the Australian freshwater turtle *Myuchelys georgesi* in February 2015. From November 2015 to November 2020, swabs were collected from turtles during repeated river surveys to estimate the prevalence of BRV RNA, identify risk factors associated with BRV infection, and refine sample collection. BRV RNA prevalence at first capture was significantly higher in *M. georgesi* (10.8%) than in a coexisting turtle, *Emydura macquarii* (1.0%). For *M. georgesi*, various risk factors were identified depending on the analysis method, but a positive BRV result was consistently associated with a larger body size. All turtles were asymptomatic when sampled and conjunctival swabs were inferred to be optimal for ongoing monitoring. Although the absence of disease and recent BRV detections suggests a reduced ongoing threat, the potential for the virus to persist in an endemic focus or resurge in cyclical epidemics cannot be excluded. Therefore, BRV is an ongoing potential threat to the conservation of *M. georgesi*, and strict adherence to biosecurity principles is essential to minimise the risk of reintroduction or spread of BRV or other pathogens.

## 1. Introduction

Serpentoviruses (frequently referred to as nidoviruses) have emerged as significant reptile pathogens worldwide [1]. Taxonomically, they are now placed within the subfamily *Serpentovirinae* (order *Nidovirales*, suborder *Tornidovirineae*, family *Tobaniviridae*) [2]. Despite now being referred to as serpentoviruses, which conveys an impression of being viruses of snakes, the subfamily *Serpentovirinae* includes multiple viruses known to infect a range of other reptiles, not just snakes. They are primarily known for causing severe and often fatal respiratory disease in captive pythons, but have also been implicated in disease events in both captive and wild populations of lizards [3,4] and turtles [5]. Respiratory disease, oral lesions, and sudden death are common features of infection in reptiles, but cases of prolonged infection and intermittent viral shedding have been reported [6,7]. These viruses have also been detected in snakes, lizards, and turtles without disease [4,5,7]. However, few studies have documented the presence of serpentoviruses in wild reptiles, and, when they have, the focus has been on invasive reptile species [7].

Bellinger River virus (BRV) was first detected in the sole extant wild population of the Bellinger River snapping turtle (*Myuchelys georgesi*) following a catastrophic mortality event in February 2015 [5]. During this event, more than 400 turtles, mainly adults, were found dead or dying over approximately seven weeks. The resulting population decline was probably more than 90%, including the loss of nearly all adults, and the persistence of the species in the wild is now in jeopardy [8]. Zhang et al. (2018) published strong indirect evidence that BRV was the principal aetiological agent causing mortality [5]. The now critically endangered wild population of *M. georgesi* also faces concurrent threats, including hybridisation with and potential competition from the locally introduced Murray River turtle (*Emydura macquarii)* and predation of nests by native goannas (*Varanus varius*) and introduced red foxes (*Vulpes vulpes*) [8,9,10,11,12]. Therefore, the recovery of the remaining wild *M. georgesi* population is a daunting prospect, and the near-total loss of mature turtles has necessitated urgent conservation actions, including the captive propagation of *M. georgesi* to maintain genetic diversity and produce viable offspring for release [8,13].

In conjunction with surveys to monitor the health, abundance, and population structure of *M. georgesi* following the mortality event, we documented the ongoing presence of BRV-infected turtles in the Bellinger River. During the survey period, from November 2015 until November 2020, the specific objectives of the study were to estimate the prevalence of BRV RNA, identify optimal sample types for ongoing monitoring, and, where possible, identify risk factors associated with BRV infection. We aimed to improve our understanding of the ongoing threat of BRV to the conservation of *M. georgesi* and add to our broader knowledge of the prevalence, dynamics, and impacts of serpentovirus infection in wild turtles. To our knowledge, it is the first study to describe the continued monitoring of a wild native reptile population after discovering a novel serpentovirus. 

## 2. Materials and Methods

### 2.1. Surveys

Samples were collected during seven turtle surveys led by NSW Department of Climate Change, Energy, the Environment, and Water (DCCEEW) in November 2015, March 2016, November–December 2016, November 2017, November 2018, November–December 2019, and November 2020. These will be referred to hereafter as routine surveys and were conducted to monitor the health, abundance, and population structure of the two freshwater turtle species inhabiting the river: *Myuchelys georgesi* and *Emydura macquarii.* Routine surveys were undertaken during late spring to summer to correspond with the naturally active phase of the two species [9,14]. Additional sampling was conducted between November 2015 and November 2020 for other purposes, such as research and assessment of turtle detectability. 

### 2.2. Study Sites

The Bellinger River is situated on the mid-north coast of New South Wales, Australia. It rises within the Great Dividing Range, southeast of Ebor, and flows unimpeded eastward for 109 km, descending over 1150 m into the Tasman Sea. It is predominantly freshwater, with a tidal influence extending approximately 20 km upstream of the mouth of the river [15]. *M. georgesi* occupies a 60 km stretch of the Bellinger River and a short section of its principal tributary, the Kalang River. 

**Figure 1:**
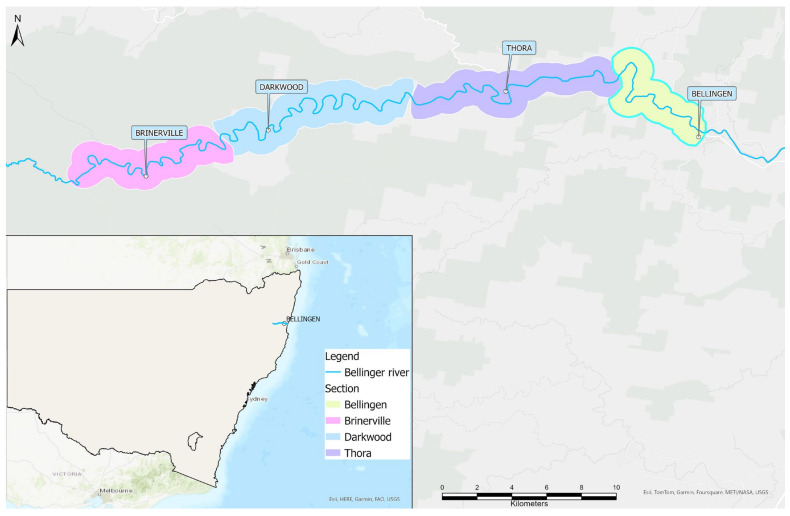
Map of the Bellinger River showing the defined river sections for the survey period.

Detailed ‘routine survey’ methodology is described by Chessman et al. (2020) [8]. Briefly, early surveys (November 2015 to November–December 2016) aimed to locate as many survivors as possible (non-randomly), with sites selected within the historical distribution of *M. georgesi* according to previous turtle observations, accessibility, and local knowledge. A survey site was generally a reach between the upstream and downstream ends of a deep pool and the river was stratified into four sections from upstream downwards: Brinerville, Darkwood, Thora, and Bellingen (Figure 1). Subsequent surveys (2017 onwards) used detailed river surveys to randomly select reaches of known or potential *M. georgesi* habitat within each section. Sites were excluded if there was fast flowing water, the site was too shallow for turtles to occupy, or if access was restricted. In these cases, nearby accessible sites were substituted. 

### 2.3. Turtle Capture and Identification

Turtles were captured mainly by divers equipped with masks, snorkels, and fins, and, occasionally, with baited cathedral traps deployed in still or slowly flowing water at approximately 1–2 m depth. Detailed capture methodology, turtle identification, and measurements are described by Chessman et al. (2020) [8]. Briefly, turtles were identified as either *M. georgesi*, *E. macquarii,* or hybrids (first filial generation: F1) of the two species by physical examination of species-specific morphologic features. Individuals were identified by unique combinations of notches in marginal scutes and, depending on turtle size, passive integrated transponder (PIT) tags. At each capture, details including date, location, species, sex if the turtle was larger than the size at which male tails enlarge (male/female/unknown), straight carapace length (mm), mass, and any abnormal physical features were recorded. 

### 2.4. Sample Size and Specimen Collection from Turtles

For each routine survey, three swabs were collected separately from the mucosal surfaces of each turtle (conjunctival, oral, and cloacal), placed in viral transport medium (3 mL of phosphate buffered gelatin saline, PBGS. pH 7.2), and held at approximately 4 °C before being sent to the laboratory. Only conjunctival swabs were collected from November 2016 and November 2019 onwards for *E. macquarii* and *M. georgesi*, respectively. To detect BRV in *M. georgesi* and *E. macquarii* populations, a sample size of 58 turtles per species per survey was required using the following formula and assumptions; n = [1 − 1 (1−p_1_) ^1/d^] [N−d/2] + 1 where N (population size) = 2000, d (minimum number of affected animals expected in the population) = 100 (5% of 2000), and p_1_ (probability of finding at least one positive in the sample) = 0.95 [16]. Unfortunately, the dramatic reduction of *M. georgesi* resulted in an unavoidable limit on the number of accessible animals. Therefore, given the uncertainty regarding changing population size and expected prevalence, samples were taken from nearly all *M. georgesi* caught at each survey. *E. macquarii* was targeted only from 2015 to 2018. Additional samples were opportunistically collected from any animal identified as an F1 hybrid (*M. georgesi* × *E. macquarii*) by physical examination. 

### 2.5. Sample Size and Specimen Collection from Other Species

Following the detection of BRV, Zhang et al. (2018) reported the results of a field survey in November 2015 (6 months after the cessation of the outbreak). This survey tested a wide range of aquatic and terrestrial animals (n = 360) and found no other species infected with BRV [5]. Additional samples from other species were collected during the March 2016 survey and were analysed in the current study. A total of 142 samples from 91 individuals, consisting of various reptiles, mammals, fishes, amphibians, and arthropods, were tested for the presence of BRV RNA. Briefly, for larger vertebrates, a single combined conjunctival (ocular) and oral mucosal swab, and, for smaller vertebrates, a skin swab was placed in viral transport medium (3 mL of phosphate-buffered gelatin saline, PBGS. pH 7.2). Samples were held at approximately 4 °C before being sent to the laboratory. Small invertebrates were preserved in absolute ethanol and prepared for nucleic extraction by first digesting in proteinase K solution as described previously [17]. Full details of the species and the numbers examined are listed in Section 3.7. 

### 2.6. Bellinger River Virus Real-Time PCR Assays

All samples were initially screened in a qRT-PCR assay targeting the sequence encoding the presumptive polyprotein 1a (replicase 1a) of BRV (BRV qRT-PCR). If a sample was positive in this assay, it was subsequently tested in an assay directed at the region encoding the ‘spike’ protein of BRV (BRV-S qRT-PCR). The primer and probe details, reaction conditions, and cycling parameters of these assays have previously been reported in Zhang et al. (2018) [5]. A turtle was considered infected if BRV RNA was detected by either the replicase 1a or spike-protein-specific assay with a cycle threshold (Ct) value ≤ 40.

### 2.7. Statistical Evaluation

Statistical analyses were performed using R Studio version 4.1.0 [18]. To explore risk factors using multivariable logistic regression several packages including gmodels [19], car [20], lmtest [21], and generalhoslem [22] were used. BRV prevalence was estimated at each survey and for the whole survey period by calculating the proportion of conjunctival swabs with a positive BRV qRT-PCR result, divided by the total number of conjunctival swabs tested. The 95% confidence interval was calculated for each estimate. The estimated prevalence for the survey period and risk factors for a positive result were explored using conjunctival swabs from first captures only (recaptures excluded). Turtles were initially analysed as a single assemblage (n = 507) and, subsequently, *M. georgesi* (n = 185) as an individual population. Detailed analysis was not performed on *E. macquarii* (n = 316) or *M. georgesi* × *E. macquarii* (n = 6) hybrid samples as individual populations. Associations between qRT-PCR result (positive/negative) and species, sex, river section, and year of capture were determined using Chi-squared (unpaired data, >5 in all groups) or Fisher’s exact tests (unpaired data, ≤5 in one group). Turtles where sex was unable to be physically determined (juveniles smaller than the size at which male tails enlarge) were recorded as of unknown sex. Associations between qRT-PCR result (positive/negative) and mass (g) and straight carapace length (SCL: mm) were determined initially with a Mann–Whitney test (unpaired data; non-normal distribution). The association of SCL and mass with the probability of having a positive BRV qRT-PCR result (odds ratio) was also calculated by logistic regression. Only turtles with SCL measurements (n = 496) were included when analysing size as a risk factor for a positive BRV result. 

Subsequently, factors were explored using a multivariate logistic regression model with an alpha of 0.05. Selection of the most parsimonious model was performed by examining turtles as a single assemblage, and then *M. georgesi* separately. Factors with few data points (six hybrid turtles) and turtle records without SCL recorded (n = 5) were removed, leaving 183 records for *M. georgesi* and 313 for *E. macquarii*. The binary response variable was defined as positive or negative for BRV, and factors were selected for inclusion in the model according to the detection of significant univariate relationships (*p* ≤ 0.25) or according to a subjective decision to include biologically interesting factors, for example, sex or size as measured by SCL. The final model specification was selected following both backward and forward stepwise methods, and the statistical significance of the contribution of individual predictors (or group of predictors) was determined. Model selection was refined using the Akaike Information Criterion (AIC), and both Wald’s test and the likelihood ratio test (LRT) were used to examine various models. Throughout the model selection, interactions between factors were explored by constructing two-interaction product terms, forcing them into the model, and examining changes in the co-efficient and *p*-values. To ensure the assumptions of the final model were met, we ensured that there were no outliers (Cook’s Distance) or multicollinearity (Variance Inflation Factor; VIF) [23]. The final model was assessed for goodness-of-fit using the Hosmer–Lemeshow test [23] and the results for the final model were presented for each predictor variable as a corrected odds ratio (OR) with 95% CI and *p*-value.

### 2.8. Longitudinal Sampling

A subset of animals (n = 139) was sampled more than once between November 2015 and November 2020. These were tested for BRV as described in Section 2.6. There was no standard interval between sampling. 

## 3. Results

### 3.1. Turtles Captured

A total of 949 samples was collected from the first capture of 507 individual turtles in the Bellinger River during routine surveys between November 2015 and November 2020 (Table 1). Multiple samples were often obtained from an individual at a single capture. Throughout the survey period, no turtle exhibited clinical signs consistent with those observed in the initial *M. georgesi* mortality event [5]. The results for all samples collected (1346 samples from 721 turtle captures), including all survey purposes, sample types, and recaptured animals between November 2015 and November 2020, can be found in the Supplementary Materials (Table S1). 

### 3.2. Viral Prevalence

Overall, BRV RNA prevalence at first capture during routine surveys was low (4.7%; 95% CI: 3.2–7.0). From November 2015 to November 2020, only 24 of the 507 individual turtles had BRV RNA detected on conjunctival swabs at first capture (Table 1). Of these, most were found in *M. georgesi* (n = 20), with a prevalence of 10.8% at first capture during routine surveys (95% CI: 7.1–16.1) (Table 1).

### 3.3. Risk Factor for a Positive Result 

Viral prevalence at first capture was significantly higher in samples from *M. georgesi* (10.8%) than in those from *E. macquarii* (1.0%) (*p* < 0.001) (Table 1). The odds of being positive for BRV RNA at first capture were 12.6 for *M. georgesi* compared to *E. macquarii* (95% CI: 3.7–43.5, *p* < 0.001). Overall, these results suggest that *M. georgesi* is significantly more susceptible to BRV infection than *E. macquarii*. BRV RNA was also detected in a single *M. georgesi* × *E. macquarii* hybrid (F1), likely leading to an unrepresentative prevalence value (16.7%) (95% CI: 3.0–56.4, n = 6) given the small number of F1 hybrids caught. Full univariate analysis on all turtle captures analysed as a single assemblage (Table S2) can be found in the Supplementary Materials; however, given the small number of detections in *E. macquarii* (n = 3) and *M. georgesi* × *E. macquarii* hybrid turtles (n = 1), further univariate analysis focused on *M. georgesi*. 

When *M. georgesi* was analysed independently, univariate analysis found a significant association between BRV result and sex, size, and river section (Table 2). For sex, viral prevalence was highest in *M. georgesi* males (38.1%), followed by females (12.5%), and then juvenile turtles of unknown sex (6.8%) (*p* < 0.001). The difference in BRV RNA prevalence was significant between males and juveniles of unknown sex (z = 4.3, *p* < 0.001), with the odds of being positive in a BRV qRT-PCR at first capture being 8.5 for male *M. georgesi* over juveniles of unknown sex (95% CI: 2.9–25.3, *p* < 0.001). In contrast, there was no significant difference in BRV RNA prevalence between females and males (z = 1.7, *p* = 0.082) or between females and juveniles of unknown sex (z = 0.8, *p* = 0.407). Throughout the survey period, BRV RNA detections at first capture were equal in adult (n = 10) and juvenile *M. georgesi* of unknown sex (n = 10) (Table 2). 

For *M. georgesi*, there was a significant association between size and BRV result (Table 2). There was a significant difference in the SCL between turtles with a positive BRV result (n = 19) and those with a negative result (n = 164) (W = 960.5, *p* = 0.006). The median SCL for *M. georgesi* with a positive result was 135.0 mm compared to 97.0 mm for a negative result. For every 1 mm increase in SCL. there was a 2% (95% CI: 0.6–3.4%) increase in the odds of a BRV positive result (*p* = 0.004). 

For *M. georgesi*, there was a significant association between the river section and BRV result (*p* = 0.019) (Table 2) with viral prevalence highest in the Thora section (16.9%), followed by the Bellingen (10.7%) section. No samples were included from the Brinerville section because of the scarcity of captures. A Kruskal–Wallis test (*H*(2) = 29.14, *p* = <0.01) showed that larger turtles were found in the Thora section (median SCL = 115.3 mm), followed by the Darkwood section (median SCL = 94.18 mm), and then the Bellingen section (median SCL = 93.3 mm). There was no BRV RNA detected in the Darkwood section throughout the survey period. The viral prevalence in the Bellingen and Thora sections did not differ significantly (z = 1.1, *p* = 0.271). However, there was a statistically significant difference between the Darkwood section and both the Bellingen (z = 2.0, *p* = 0.041) and Thora (z = 2.6, *p* = 0.009) sections (Table 2). 

### 3.4. Multivariate Logistic Regression Model

A subset (n = 496) of the 507 individual turtles caught for molecular testing at first capture was included in multivariate analysis as a single assemblage and included in Supplementary Materials (Table S3). Subsequently, a subset of *M*. *georgesi* (n = 183) was analysed independently. The final model, using *M. georgesi* only, was selected using a backward stepwise method and adjusted odds ratios were obtained (Table 3). Mass was excluded from the final model because of multicollinearity with SCL. There was a significant interaction between SCL and year of capture and this interaction was then adjusted in the model. The results were non-significant for sex and river section. For the final multivariable model, the Hosmer–Lemeshow test showed that the model fitted the data (*p* = 0.853). 

**Figure 2:**
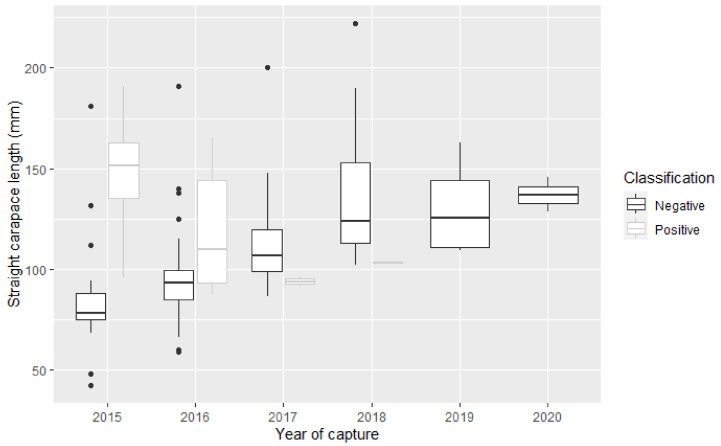
Boxplot showing the relationship between straight carapace length and year of capture for *M. georgesi* included in the multivariate logistic regression model. The proportion of *M. georgesi* with BRV RNA detected (positive; grey), the total number captured, and the estimated prevalence varied in 2015 (n = 8/29; estimated prevalence: 27.6%), 2016 (n = 7/87; estimated prevalence 8.0%), 2017 (n = 3/31; estimated prevalence 9.7%), 2018 (n = 1/26; estimated prevalence 3.8%), 2019 (n = 0/8; estimated prevalence 0%), and 2020 (n = 0/2; estimated prevalence 0%).

According to the final model, there was a significant relationship between SCL and a positive BRV result (*p* = 0.017) (Table 3). For every 1 mm increase in SCL, there was a 9% increase in the odds of a positive BRV result (95% CI: 2–19). The model also highlighted a significant reduction in risk for samples collected in 2017 (OR: 0.83; 95% CI: 0.066–0.93). The interaction between SCL and year of capture is highlighted in Figure 2, where the association of larger *M. georgesi* and a positive BRV result is most evident in 2015 and 2016. In 2017 and 2018, BRV-infected *M. georgesi* have a smaller SCL than their negative counterparts. 

### 3.5. Longitudinal Sampling

A subset of animals (n = 139) was sampled more than once during the survey period. Of these, 18 *M. georgesi* had BRV detected at least once (Table 4), four of them transitioning from positive to consistently negative (≥2 subsequent negative results). BRV RNA was also detected at a single time point in one *E. macquarii* in the lower section (Bellingen) at first capture, with a subsequent negative result (Table 4). Throughout the survey period, no individual *E. macquarii* or hybrid *M. georgesi* × *E. macquarii* had BRV detected at more than one capture.

BRV RNA was detected more than once in seven *M. georgesi*, most of which were juveniles of unknown sex. The longest time between two consecutive BRV RNA detections was 736 days (2.02 years) (MG4—Table 4). BRV was detected at the second capture after a negative result in three juvenile *M. georgesi*, all captured in 2016. These three turtles were not recaptured. BRV RNA was detected at a single time point in 8 *M. georgesi* at either their first or second capture with subsequent negative results. Of these, all were juvenile except for a single adult male that was not captured again following the negative result.

Most turtles sampled more than once always had negative BRV qRT-PCR results: 49 *M. georgesi*, 68 *E. macquarii*, and 3 hybrids. These turtles tested negative two to four times with an average of 477, 602, and 601 days between the first and last sampling for *M. georgesi*, *E. macquarii,* and hybrids, respectively. At a site within the Bellingen section, 7 *M. georgesi* were consistently negative although conspecifics at the same location had positive results.

### 3.6. Refining Sample Collection

A subset of 301 turtles was screened for BRV with all three swab types: conjunctival, oral, and cloacal, from November 2015 to November 2020 (Table 5). Conjunctival swabs were the most sensitive sample type, resulting in the most BRV RNA detections, followed by oral, and, finally, cloacal swabs. Notably, an oral or cloacal swab was never positive without a positive conjunctival swab from the same animal.

In contrast to the 2015 outbreak, these animals were asymptomatic, and mostly low levels of viral RNA were detected in any animal throughout this period, with only a single animal with a Ct value < 30 (Table 6). 

### 3.7. Results from Other Species

The results for all species other than turtles that were tested in the BRV qRT-PCR assay were negative (n = 94; 95% CI; 0–3.9) (Table 7).

## 4. Discussion

This study documents the presence of BRV-infected turtles in the Bellinger River between November 2015 and November 2020. The overall survey estimated prevalence of BRV RNA was low (4.7%), and BRV RNA was not detected beyond November 2018. Throughout the study, no turtle exhibited clinical signs consistent with those observed in the initial *M. georgesi* mortality event. The gradual reduction in BRV prevalence over the survey and the absence of ongoing disease appear consistent with the tail end of an epidemic. However, it is rare for epidemics to end suddenly. Instead, they more often persist as cyclical epidemics or endemic diseases [24]. Unfortunately, given the dramatic reduction in the number of *M. georgesi* captured from 2018 onwards, the possibility of an ongoing low-level BRV presence cannot be excluded.

The reduction in the number of *M. georgesi* captured could be influenced by shifts in the survey methodology and variability in environmental conditions, or could represent undetected ongoing mortalities. The shift from a non-random to a more randomised approach may have influenced both the captures of *M. georgesi* and estimates of viral prevalence. River height, water temperature, flow, and underwater visibility also varied during the surveys [8]. These changes followed seasonal variations in air temperature, storms of varying severity, and bushfires in November 2019, all of which may have had an impact on turtle captures. Nevertheless, there is consistent evidence of an ongoing decline in the abundance of *M. georgesi* as documented previously [8]. In contrast, there has been no evidence of impact on the *E. macquarii* population, suggesting the impact is species-specific. The most recent estimate of the remaining wild *M. georgesi* population, ~150 individuals, highlights the dramatic reduction since the mortality event, especially when compared to historical estimates of between ~2200 [25] and ~4500 [10]. While ongoing mortalities due to BRV were not documented during the surveys, the possibility cannot be excluded given the rarity of *M. georgesi* and the length of the river it occupies. 

The mortality event that *M. georgesi* suffered in 2015 was comparable to mass mortality of amphibians infected with chytrid fungus (*Batrachochytrium dendrobatidis* and *Batrachochytrium salamandrivorans*) [26,27] and was far more significant than other reported disease outbreaks in Australian freshwater turtles [28,29,30,31]. This severity can be contrasted with the low overall BRV prevalence found in this study. This study is unique in this field as the first to describe serpentovirus prevalence in wild turtle populations, with other findings for wild reptiles limited to a single observation of a prevalence of 24.4% in wild invasive Burmese pythons (*Python bivittatus*) (n = 172) [7]. This prevalence is similar to that observed in November 2015, but the overall low BRV prevalence in the present study may be influenced by survivorship bias, with the turtles surveyed having an uncertain BRV exposure status before the study. Surviving the 2015 mortality event could have resulted from a lack of virus exposure, but, more likely, a successful innate or acquired immune response or higher individual resistance, both of which may have influenced BRV prevalence in this study. Additionally, the utilised PCR targeted a conserved region of the BRV genome to detect BRV specifically [5]. Therefore, we cannot exclude the presence of other serpentoviruses in any species sampled as part of this study. Developing additional broadly reactive diagnostic assays and metagenomic sequencing of negative samples could minimise this possibility and is an opportunity for future research [32]. 

In contrast to the 2015 mortality event, all turtles in this study were asymptomatic at the time of BRV RNA detection [5]. Asymptomatic animals have also been reported in other serpentovirus-infected reptile species [6,7,33,34]. Testing during the incubation period, true asymptomatic infection, or recovery but in a persistent carrier state could explain such results. Longitudinal sampling found BRV-infected *M. georgesi* that were not recaptured in subsequent surveys, had subsequent negative results, or had BRV detected again much later. Only a few *M. georgesi* had two consecutive BRV RNA detections, and the longest time between detections was over two years. Hypothesising outcomes for BRV-infected turtles not recaptured and differentiating between persistent or chronic infections and re-infection is challenging when monitoring free-ranging turtles. The interpretation of subsequent negative results, potentially occurring as a result of clearing BRV infection, is also complicated by the low levels of virus found on conjunctival swabs and the potential for testing at the limit of detection or intermittent viral shedding, a possibility indicated by research on serpentovirus-infected captive pythons monitored at four-month intervals for over two years [6]. Detecting asymptomatic BRV-infected turtles is an important consideration for the development and implementation of biosecurity protocols for captive freshwater turtle collections and conservation programs. It also highlights the need for additional research into the conditions required to cause clinical BRV disease in these turtles.

The apparently long duration of infection in some turtles facilitates PCR-based monitoring for BRV, especially in the absence of a serological assay to determine previous exposure. Persistent or chronic serpentovirus infection in asymptomatic reptiles has been reported in other species, including snakes [6,7] and lizards [3]. Co-infections with two genetically diverse serpentoviruses have also been reported in both snakes and lizards. However, clear re-infection with the same serpentovirus after apparent recovery has yet to be reported [3,6]. Serially collected samples have the potential to support nucleic acid sequencing studies that may allow the differentiation between persistent or chronic infection and re-infection in wild turtles. Conversely, the uncertainties raised by asymptomatic infections, intermittent shedding, and varying viral loads highlight the need for additional diagnostic options, including exploring the development and use of serological assays.

Zhang et al. (2018) reported an initial detection of BRV in two *E. macquarii* in November 2015 [5]. Subsequently, the present study found only one more detection in *E. macquarii* in December 2016, again at low levels. BRV was not detected in any further *E. macquarii* despite many samples. BRV RNA has been detected in a single F1 hybrid of *M. georgesi* × *E. macquarii*, but few F1 hybrids were sampled in this survey and evaluating their susceptibility is, therefore, difficult. In this study, the limited detections of BRV RNA in *E. macquarii* suggest that either the opportunity for transmission of BRV did not occur, only transient infection occurred, or that *E. macquarii* may have lower susceptibility to BRV infection. In any case, it is unlikely that *E. macquarii* acts as a reservoir for ongoing BRV transmission. Differences in serpentovirus prevalence among species have also been reported in snakes [6,33,35,36] and lizards [3]. Collectively, for BRV and other serpentoviruses, the apparent differences in prevalence among species highlight the need for further research into the factors contributing to both infection and disease, including the use of genomics to highlight immune gene variability among host species, continued viral metagenomics to provide the foundation for determining host-specific serpentovirus lineages, and transmission trials to explore the susceptibility of a range of species. 

Several factors were associated with a positive BRV result when *M. georgesi* was analysed independently of *E. macquarii*, including sex, size, and river section. Male *M. georgesi* had a significantly higher prevalence of BRV infection than juveniles of unknown sex in univariate analysis. However, the number of adult *M. georgesi* available limited our ability to find significant differences between male and female turtles. The significant difference in prevalence between male *M. georgesi* and juveniles of unknown sex could be confounded by differences in the size of the turtles, which may be why sex was not selected as a risk factor in the final multivariate logistic regression model. Differences in serpentovirus prevalence between sexes have been reported in pythons [7], with males having a higher viral prevalence than females. The differences in prevalence between males and juveniles of unknown sex could be related to their reproductive status, age-related differences in immunity, or differences in behaviour that could potentially influence both exposure and viral transmission rates. 

A larger size, measured by SCL, was also associated with a positive BRV result by both univariate analysis and the final multivariate logistic regression model. The association of larger *M. georgesi* and a positive BRV result was most evident in 2015 and 2016, whereas, in 2017 and 2018, positive *M. georgesi* had a smaller SCL. This finding is consistent with mostly adult turtles succumbing to the mortality event, and approximately 88% of survivors being immature [8]. Previous authors have hypothesised age-related differences in diet or behaviour as contributing factors for BRV exposure, or other unknown differences in susceptibility between adult and juvenile turtles [8,25]. The apparently greater susceptibility of older turtles contrasts with a wide range of plant and animal studies typically finding juveniles more susceptible to disease than adults [37,38,39,40,41]. There are, however, other exceptions for this generalisation; for example, pythons infected with a serpentovirus tended to be larger than non-infected ones [6,7]. It is possible that the association of a positive result with males compared to juveniles, and with larger *M. georgesi*, could be much more significant than suggested by the present findings, given the paucity of available adult *M. georgesi* over the survey period. This finding is also an important consideration as the remaining, mostly juvenile wild *M. georgesi* population ages over time.

BRV prevalence in *M. georgesi* also differed significantly among river sections, though not when other risk factors were considered concurrently as part of the final multivariate logistic regression model. A likely explanation for this finding is that the size of turtles, as measured by SCL, was not uniform across the Darkwood, Thora, and Bellingen river sections, with larger turtles found in the Thora section. Throughout the surveys, there were no detections in the Darkwood (upper) section, compared to several in the Thora (middle) and Bellingen (lower) river sections. This pattern can be contrasted with the reporting of diseased turtles throughout the river system during the 2015 outbreak [42]. In addition to the known differences in turtle size between the river sections, the higher prevalence in some sections could also reflect historical turtle densities, differences in mortality rates between river sections, or locations of turtles that may have a chronic or persistent infection. 

Conjunctival swabs resulted in the most BRV RNA detections in asymptomatic animals, and are, therefore, the recommended sample type for ongoing monitoring. Furthermore, in this study, only low levels of viral RNA were detected in asymptomatic turtles, except for a single sample with moderate amounts. This finding can be contrasted with results for samples collected from clinically affected animals at the time of the outbreak, where viral RNA was detected on all three swab types, generally with high virus loads [5]. Furthermore, in situ hybridisation was used to confirm the presence of viral nucleic acid within a severely affected lacrimal gland from a clinically affected animal at the time of the outbreak [5]. Despite the benefits of using this technology on asymptomatic BRV-infected turtles to provide insight into the pathophysiology of persistent or chronically infected turtles, and perhaps why conjunctival swabs resulted in the highest number of BRV detections, the critically endangered status of *M. georgesi* limits such investigation. 

Finally, the origin of BRV is yet to be determined. There was no evidence of virus in species other than turtles sampled from the affected area in November 2015 [5] or the current study. At the time of its identification, BRV was the first serpentovirus associated with disease in an aquatic reptile. Since then, the study of serpentoviruses, while initially focused on research in captive python populations, has expanded to the discovery of more divergent serpentoviruses in a broader range of reptile species. Despite this development, the prevalence and distribution of these viruses in wild reptile populations remains largely unknown. 

In conclusion, this study has estimated BRV prevalence in the Bellinger River following the 2015 mortality event, refined sample collection, and identified risk factors associated with BRV infection. Key aspects of the epidemiology of BRV have been revealed, specifically the potential for this virus to persist in asymptomatic animals, as in serpentovirus infections in snakes and lizards. This finding will be an essential consideration for developing and implementing biosecurity protocols for captive freshwater turtle collections and conservation programs. While the absence of both clinical disease and recent detections of BRV suggests a reduced ongoing threat to the conservation of *M. georgesi*, the potential of BRV to persist in an endemic focus or resurge in cyclical epidemics cannot be excluded. The wild *M. georgesi* population is expected to grow with conservation efforts, and the proportion of adult turtles increase. However, in the absence of robust data to identify the origin of BRV, serpentoviruses must be considered an ongoing threat to the already critically endangered population of *M. georgesi.* Consequently, it is essential that appropriate biosecurity principles and protocols must be applied to minimise risks of a reintroduction and spread of BRV or, indeed, other pathogens.

## Figures and Tables

**Table 1:** Numbers of sampled wild turtles and sample types along with the number of BRV qRT-PCR positive samples (+).

Survey	No. Turtles Caught	No. Turtles for Molecular Testing First Capture * (BRV RNA +)	Estimated Prevalence % (95% CI)	Conjunctival Swab (BRV RNA +)	Oral Swab (BRV RNA +)	Cloacal Swab (BRV RNA +)	Total Swabs (BRV RNA +)
*Myuchelys georgesi*							
November 2015 ^#^	29	29 (8)	27.6 (14.7–45.7)	29 (8)	15 (2)	18 (0)	62 (10)
March 2016	56	45 (2)	4.4 (1.2–14.8)	45 (2)	39 (0)	45 (0)	129 (2)
November/December 2016	63	43 (6)	14.0 (6.6–27.3)	43 (6)	0 (0)	0 (0)	43 (6)
November 2017	39	31 (3)	9.7 (3.4–24.9)	31 (3)	19 (2)	25 (0)	75 (5)
November 2018	43	27 (1)	3.7 (0.6–18.3)	27 (1)	26 (0)	27 (0)	80 (1)
November/December 2019	26	8 (0)	0 (0–32.4)	8 (0)	0(0)	0 (0)	8 (0)
November 2020	13	2 (0)	0 (0–65.8)	2 (0)	0 (0)	0 (0)	2 (0)
**Total:**	**269**	**185 (20)**	**10.8 (7.1–16.1)**	**185 (20)**	**99 (4)**	**115 (0)**	**399 (24)**
*Emydura macquarii*							
November 2015 ^#^	49	49 (2)	4.1 (1.1–13.7)	49 (2)	43 (0)	43 (0)	135 (2)
March 2016	89	70 (0)	0 (0.0–5.2)	70 (0)	69 (0)	69 (0)	208 (0)
November/December 2016	69	47 (1)	2.13 (0.4–11.1)	47 (1)	0 (0)	0 (0)	47 (1)
November 2017	169	72 (0)	0 (0–5.07)	72 (0)	0 (0)	0 (0)	72 (0)
November 2018	149	52 (0)	0 (0–6.9)	52 (0)	0 (0)	0 (0)	52 (0)
November/December 2019	130	26 (0)	0 (0–12.9)	26 (0)	1 (0)	0 (0)	26 (0)
November 2020	118	0 (0)		0 (0)	0 (0)	0 (0)	0 (0)
**Total:**	**773**	**316 (3)**	**1.0 (0.3–2.8)**	**316 (3)**	**113 (0)**	**112 (0)**	**540 (3)**
*Myuchelys georgesi* x *Emydura macquarii* (F1)							
March 2016	1	1 (0)	0 (0–79.4)	1 (0)	1 (0)	1 (0)	3 (0)
November/December 2016	3	3 (1)	33.3 (6.2–79.2)	3 (1)	0 (0)	0 (0)	3 (1)
November 2017	3	1 (0)	0 (0–79.4)	1 (0)	1 (0)	1 (0)	3 (0)
November 2020	2	1 (0)	0 (0–79.4)	1 (0)	0 (0)	0 (0)	1 (0)
**Total:**	**9**	**6 (1)**	**16.7 (3.0–56.4)**	**6 (1)**	**2 (0)**	**2 (0)**	**10 (1)**
**All turtle species**	**1076**	**507 (24)**	**4.7 (3.2–7.0)**	**507 (24)**	**214 (4)**	**229(0)**	**949 (28**)

Results for turtles caught during ‘routine surveys’ only. Results of recaptured turtles were not included in this table. BRV RNA detected (BRV RNA +). Turtles for molecular testing at first capture included if conjunctival (ocular) swab collected (*). Data previously reported in Zhang et al. (2018) [5] (^#^).

**Table 2:** Epidemiological data for wild *M. georgesi* turtles captured in the Bellinger River from November 2015 to November 2020—univariate analysis.

Variable	Categories	N	Positive (%; 95% CI)	Negative	*p*	df
Sex	Unknown	148	10 (6.8; 3.7–12.0)	138	**<0.001** *	2
	Female	16	2 (12.5; 3.5–36.0)	14		
	Male	21	8 (38.1; 20.8–59.1)	13		
River section	Bellingen	84	9 (10.7; 5.7–19.1)	75	0.019 *	2
	Thora	65	11 (16.9; 9.7–27.8)	54		
	Darkwood	36	0 (0; 0–9.6)	36		
Year	2015	29	8 (27.6; 14.7–45.7)	21	0.093 *	5
	2016	88	8 (9.1; 4.7–16.9)	80		
	2017	31	3 (9.7; 3.4–24.9)	28		
	2018	27	1 (3.7; 0.7–18.3)	26		
	2019	8	0 (0; 0–32.4)	8		
	2020	2	0 (0; 0–65.8)	2		
**Variable**	**Categories**	**N**	**Positive (IQR)**	**Negative (IQR)**	***p***	**df**
Size	SCL (mm)	183	135.0 (60.2)	97.0 (26.5)	**0.006** ^#^	182

Results for turtles caught during ‘routine surveys’ only. Results of recaptured turtles were not included in this table. Statistically significant = **BOLD**, * Fisher’s Exact Test, ^#^ Mann–Whitney test. SCL reported as median and interquartile range (IQR) for each positive and negative turtle groups.

**Table 3:** Best fit multivariate logistic regression model for selected risk factors associated with a positive BRV qRT-PCR result for *M. georgesi*.

Factors	Categories	Corrected OR (95% CI)	*p*	AIC
SCL (mm)	Negative			98.31
	Positive	1.09 (1.02–1.19)	**0.017**	
Sex	Unknown			
	Female	0.01 (0.00–1.88)	0.122	
	Male	1.49 (0.04–66.63)	0.831	
Location	Bellingen			
	Thora	0.48 (0.08–2.11)	0.366	
	Darkwood	0.00	0.992	
Year * SCL	2015			
	2016	0.97 (0.91–1.02)	0.334	
	2017	0.83 (0.66–0.93)	**0.027**	
	2018	0.54 (0.02–0.80)	0.393	
	2019	0.93 *	1.000	
	2020	1.21 *	1.000	

Results for turtles caught for the first time during ‘routine surveys’ and those with a straight carapace length (SCL) recorded were included (n = 183) in the model. Odds ratio (OR). Akaike Information Criterion (AIC). * 95% CI not calculated.

**Table 4:** Longitudinal BRV qRT-PCR results for individual turtles with BRV detected at one or more sampling points between November 2015 and November 2020.

	Month of Capture															
	2015	2016			2017		2018		2019	2020						
Turtle	Nov	Mar	Nov	Dec	Feb	Nov	Apr	Nov	Nov	Nov	Days ^a^	Sex	Location	SCL (mm) ^b^	Δ SCL (mm) ^c^	Δ Mass (grams) ^d^
	*Myuchelys georgesi*—BRV RNA detected at two time points															
MG1	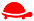	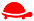									115	U	T	135.2	4.2	20
MG2		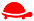	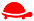								265	M	T	153.5	−0.4	42.5
MG3	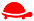	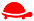			**NC**		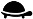			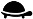	1827	F	T	190.8	8.6	197
^#^ MG4		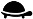	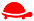					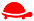			990	U	B	89.7	15.9	49
MG5							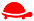	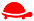			237	U	B	101.0	2.0	12
MG6						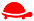	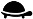	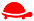			379	U	B	93.9	12.2	33
MG7						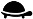	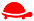	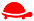	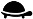		736	U	B	105.7	17.0	60.9
	**Average:**										**650**			**124.3**	**8.5**	**59.2**
	*Myuchelys georgesi*—BRV RNA detected at a single time point															
MG8		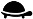	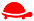								256	U	B	107.3	2.8	6.50
MG9		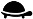	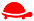								255	U	B	89.9	4.1	11.50
MG10		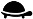	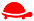								255	U	B	69.3	7.8	13.00
	**Average:**										**255**			**88.8**	**4.9**	**10.3**
	*Myuchelys georgesi*—BRV RNA detected at a single time point with subsequent negative results															
MG11	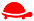	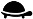									118	U	T	135.4	5.8	21
MG12	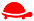	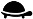									118	U	T	95.8	10.5	36
MG13	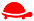	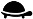									118	M	T	145.9	−1.0	−4.5
MG14		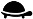				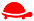	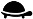	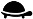	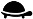		1349	U	B	93.3	24.3	85.2
MG15		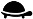	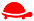				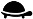				756	U	B	93.3	21.2	NR
MG16		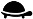	**NC**			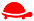	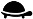	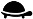			992	U	B	59.0	41.1	94
MG17						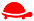	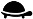	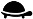	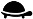	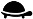	1095	U	B	96.6	19.0	80
MG18						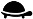		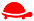	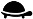		736	U	B	88.7	12.5	52
	**Average:**										**660**			**101**	**16.7**	**52**
	*Emydura macquarii*—BRV RNA detected at a single time point															
EM1	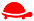	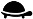	**NC**			**NC**					732	F	B	160.3	18.2	198.5

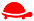
 BRV RNA detected on a conjunctival swab; 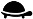 BRV RNA not detected on a conjunctival swab; Days between first and last capture (^a^); Sex (male (M), female (F), unknown (U)); Location (Thora (T), Bellingen (B)); Straight carapace length (SCL) (mm) at first capture (^b^); Change (Δ) in SCL (mm) between first and last sampling (^c^); Change (Δ) in mass (g) between first and last sampling (^d^); captured but no sample collected (NC); Turtle with longest time between two consecutive BRV RNA detections (#). Results for individual turtles caught during routine surveys and additional research surveys. Results of recaptured turtles with negative BRV q-RT-PCR results at all sampling points were not included in this table.

**Table 5:** Comparison of three swab types (conjunctival, oral, and cloacal) for turtles (n = 301) screened with BRV qRT-PCR.

Survey	Conjunctival Swab (BRV RNA +)	Oral Swab (BRV RNA +)	Cloacal Swab (BRV RNA +)
*Myuchelys georgesi*			
November 2015	15 (8)	15 (2)	15 (0)
March 2016	52 (4)	52 (0)	52 (0)
November 2017	28 (5)	28 (5)	28 (1)
April 2018	11 (0)	11 (0)	11 (0)
November 2018	45 (4)	45 (0)	45 (0)
March/April 2019	4 (0)	4 (0)	4 (0)
Total:	155 (21)	155 (7)	155 (1)
*Emydura macquarii*			
November 2015	43 (2)	43 (0)	43 (0)
March 2016	100 (0)	100 (0)	100 (0)
April 2018	1 (0)	1 (0)	1 (0)
Total:	144 (2)	144 (0)	144 (0)
*Myuchelys georgesi* × *Emydura macquarii* (F1)			
March 2016	1 (0)	1 (0)	1 (0)
November 2017	1 (0)	1 (0)	1 (0)
Total:	2 (0)	2 (0)	2 (0)

BRV RNA detected (BRV RNA +).

**Table 6:** Comparison of three swab types (conjunctival, oral, and cloacal) for turtles with BRV RNA detected.

Turtle	Date	Conjunctival Swab (Ct Value)	Oral Swab (Ct Value)	Cloacal Swab (Ct Value)
*Myuchelys georgesi*				
MG-A	November 2015	35.29	38.14	Negative
MG-B		33.51	Negative	Negative
MG-C		35.56	Negative	Negative
MG-D		31.71	Negative	Negative
MG-E		38.01	Negative	Negative
MG-F		32.31	Negative	Negative
MG-G		34.32	Negative	Negative
MG-H		38.12	38.36	Negative
MG-I	March 2016	32.01	Negative	Negative
MG-J		33.26	Negative	Negative
MG-K		33.48	Negative	Negative
MG-L		33.67	Negative	Negative
MG-M	November 2017	35.67	37.58	Negative
MG-N		33.55	34.37	Negative
MG-O		36.32	38.86	Negative
MG-P		32.58	36.12	38.1
MG-Q		37.61	38.35	Negative
MG-R	November 2018	**29.86**	Negative	Negative
MG-S		34.73	Negative	Negative
MG-T		32.52	Negative	Negative
MG-U		33.12	Negative	Negative
**Average Ct value:**		**34.15**	**37.40**	**N/A**
*Emydura macquarii*				
EM-A	November 2015	37.34	Negative	Negative
EM-B		36.95	Negative	Negative
	**Average Ct value:**	**37.15**	**N/A**	**N/A**

Positive BRV qRT-PCR results reported as cycle threshold (Ct) value.

**Table 7:** Other species sampled from the Bellinger River March 2016 field survey.

Class	Species	Animals Tested	BRV RNA Detected
Reptilia	*Intellagama lesueurii lesueurii* (Eastern water dragon)	1	0
	*Morelia spilota* (Carpet python)	1	0
Actinopterygii	*Gobiomorphus coxii* (Cox’s gudgeon)	2	0
	*Tandanus bellingerensis* (Bellinger catfish)	8	0
	*Gambusia holbrooki* (Eastern gambusia)	7	0
	*Anguilla reinhardtii* (Long finned eel)	29	0
	*Macquaria novemaculeata* (Australian bass)	2	0
	*Potamalosa richmondia* (Freshwater herring)	2	0
	*Melanotaenia duboulayi* (Crimson-spotted rainbowfish)	1	0
Amphibia	*Mixophyes* sp. (Tadpoles–barred frogs)	3	0
	Unknown species (Tadpoles)	3	0
	*Australatya striolata* (Riffle shrimp)	6	0
Malacostraca	Orthoptera (Katydids)	9	0
Insecta	Unknown species (Mayfly larvae)	1	0
Bivalvia	Unknown species (Mussels)	1	0
Gastropoda	Unknown species (Snails)	1	0
Clitellata	Unknown species (Hirudinea–leech)	9	0
Mammalia	*Pteropus poliocephalus* (Grey-headed flying fox)	4	0
	*Ornithorhynchus anatinus* (Platypus)	1	0
**Total:**		**91**	**0**

## Data Availability

The data are contained within the article and Supplementary Materials.

## Supplementary Materials

The following supporting information can be downloaded at: https://www.mdpi.com/article/10.3390/v16040653/s1, Table S1: Numbers of sampled wild turtles caught for all purposes including recaptures along with the number of BRV RNA reactor samples (+) in the Bellinger River in NSW; Table S2: Epidemiological data for wild turtles captured in the Bellinger River, November 2015–November 2020—univariate analysis; Table S3: Epidemiological data for wild turtles captured in the Bellinger River as a single assemblage, November 2015–November 2020—univariate analysis and multivariate logistic regression model.
