# Supplementary material for: Delving into the Aftermath of a Disease-Associated Near-Extinction Event: A Five-Year Study of a Serpentovirus (Nidovirus) in a Critically Endangered Turtle Population
Source: Viruses. 2024 Apr 22;16(4):653. doi: 10.3390/v16040653 (PMC11055124; doi:10.3390/v16040653)
Supplement: Supplementary file 1 [file viruses-16-00653-s001.zip › Table S1.pdf]

Table S1. Numbers of sampled wild turtles caught for all purposes including recaptures along with the number of BRV RNA reactor samples (+) in the Bellinger River in NSW

Survey	No. turtles caught	No turtles for molecular testing (BRV RNA +[a])	Prevalence % (95%CI)	Conjunctival swab (BRV RNA +)	Oral swab (BRV RNA +)	Cloacal swab (BRV RNA +)	No ID swab (BRV RNA +)	Total samples (BRV RNA +)
Myuchelys georgesi								
November 2015[b]	29	29 (8)	27.6 (14.7-45.7)	29 (8)	15 (2)	18 (0)	11 (0)	73 (10)
March 2016	69	58 (4)	6.9 (2.7-16.4)	58 (4)	52 (0)	58 (0)	4 (0)	172 (4)
October/November/December 2016	73	61 (11)	18.0 (10.4-29.5)	61 (11)	0 (0)	0(0)	0(0)	61 (11)
February 2017	14	0 (0)	N/A	0 (0)	0 (0)	0 (0)	0 (0)	0 (0)
November 2017	43	41 (5)	12.2 (5.3-25.5)	41 (5)	28 (5)	36 (1)	0 (0)	105 (11)
April 2018	25	18 (2)	11.1 (3.1-32.8)	18 (2)	11 (0)	18 (0)	0 (0)	47 (2)
November 2018	51	49 (5)	10.2 (4.4-21.8)	49 (5)	45 (1)	49 (0)	0 (0)	143 (6)
March/April 2019	6	6 (0)	0 (0-39.0)	6 (0)	4 (0)	6 (0)	0 (0)	16 (0)
November/December 2019	29	25 (0)	0 (0-13.3)	25 (0)	1 (0)	1 (0)	0 (0)	27 (0)
October/November 2020	13	12 (0)	0 (0-24.3)	12 (0)	0 (0)	0 (0)	0 (0)	12 (0)
**Total:**	**352**	**299 (35)**	**11.7 (8.5-15.8)**	**299 (35)**	**156 (8)**	**186 (1)**	**15 (0)**	**656 (44)**
Emydura macquarii								
November 2015[b]	49	49 (2)	4.1 (1.1-13.7)	49 (2)	43 (0)	43 (0)	0 (0)	135 (2)
March 2016	100	87 (0)	0 (0-4.2)	87 (0)	86 (0)	86 (0)	0 (0)	259 (0)
November/December 2016	71	57 (1)	1.8 (0.3-9.3)	57 (1)	0 (0)	0 (0)	0 (0)	57 (1)
February 2017	4	0 (0)	N/A	0 (0)	0 (0)	0 (0)	0 (0)	0 (0)
November 2017	176	97 (0)	0 (0-3.8)	97 (0)	0 (0)	0 (0)	(0)	97 (0)
April 2018	13	1 (0)	0 (0-79.4)	1 (0)	1 (0)	1 (0)	0 (0)	3 (0)
November 2018	153	72 (0)	0 (0-5.1)	72 (0)	0 (0)	0 (0)	0 (0)	72 (0)
March/April 2019	24	9 (0)	0 (0-29.9)	9 (0)	0 (0)	0 (0)	0 (0)	9 (0)
November/December 2019	132	41 (0)	0 (0-8.6)	41 (0)	1 (0)	0 (0)	0 (0)	42 (0)
November 2020	166	0 (0)	N/A	0 (0)	0 (0)	0 (0)	0 (0)	0 (0)
**Total:**	**888**	**413 (3)**	**0.7 (0.3-2.1)**	**413(3)**	**131 (0)**	**130 (0)**	**0 (0)**	**674 (3)**
Myuchelys georgesi x *Emydura macquarii* (F1)								
March 2016	1	1 (0)	0 (0-79.4)	1 (0)	1 (0)	1 (0)	0 (0)	3 (0)
November/December 2016	3	3 (1)	33.3 (6.2-79.2)	3 (1)	0 (0)	0 (0)	0 (0)	3 (1)
November 2017	3	3 (0)	0 (0-56.2)	3 (0)	3 (0)	2 (0)	0 (0)	8 (0)
November 2020	2	2 (0)	0 (0-65.8)	2 (0)	0 (0)	0 (0)	0 (0)	2 (0)
**Total:**	**9**	**9 (1)**	**11.1 (2.0-43.5)**	**9 (1)**	**4 (0)**	**3 (0)**	**0 (0)**	**16 (1)**
**All turtle species**	**1249**	**721 (39)**	**5.4 (4.0-7.3)**	**721 (39)**	**291 (8)**	**319 (1)**	**15 (0)**	**1346 (48)**

Results of turtles caught for all purposes including surveys, additional research, survey method validation. Results of recaptured turtles were included in this table. Turtles were reactive for BRV RNA on a conjunctival (ocular) swabs (*). Data previously reported in Zhang et al (2018) ([#]). N/A – not applicable
