# Supplementary material for: Delving into the Aftermath of a Disease-Associated Near-Extinction Event: A Five-Year Study of a Serpentovirus (Nidovirus) in a Critically Endangered Turtle Population
Source: Viruses. 2024 Apr 22;16(4):653. doi: 10.3390/v16040653 (PMC11055124; doi:10.3390/v16040653)
Supplement: Supplementary file 1 [file viruses-16-00653-s001.zip › Table S2.pdf]

Table S2. Epidemiological data for all wild turtles captured in the Bellinger River, November 2015–November 2020 – univariate analysis.

**Variable**	**Categories**	**N**	**Positive (%;95% CI)**	**Negative**	***p***	**df**
Species	*E. macquarii*	313	3 (1.0; 0.3-2.8)	316	**<0.001***	2
	M. georgesi	185	20 (10.8; 7.1-16.1)	165		
	Hybrid	6	1 (16.7; 3.0-56.4)	5		
Sex	Unknown	235	10 (4.3; 2.3-7.7)	225	0.451^	2
	Female	137	5 (3.7; 1.6-8.3)	132		
	Male	135	9 (6.7; 3.6-12.2)	126		
Location	Bellingen	292	11 (3.8; 2.1-6.6)	281	**<0.001***	2
	Thora	115	13 (11.3; 6.7-18.4)	102		
	Darkwood	100	0 (0; 0-3.7)	100		
Year	2015	78	10 (12.8; 7.1-22.0)	68	**0.020***	5
	2016	209	10 (4.8-2.6-8.6)	199		
	2017	104	3 (2.9; 0.1-8.1)	101		
	2018	79	1 (1.3; 0.2-6.8)	78		
	2019	34	0 (0-9.0)	34		
	2020	3	0 (0-56.2)	3		
**Variable**	**Categories**	**N**	**Positive**	**Negative**	***P***	**df**
Size	SCL (mm)	501	135.3 (63.3)	138.0 (71.3)	0.428#	500
	Mass (grams)	499	300.0 (312.0)	288.3 (420.8)	0.742#	498

Results for turtles caught during 'routine surveys' only. Results of recaptured turtles were not included in this table. Statistically significant = **BOLD**, * Fisher's Exact Test, ^ Chi-Square Test, #Mann-Whitney test. SCL (mm) and mass (g) reported as median and interquartile range (IQR) for each positive and negative turtle group
