# Supplementary material for: Delving into the Aftermath of a Disease-Associated Near-Extinction Event: A Five-Year Study of a Serpentovirus (Nidovirus) in a Critically Endangered Turtle Population
Source: Viruses. 2024 Apr 22;16(4):653. doi: 10.3390/v16040653 (PMC11055124; doi:10.3390/v16040653)
Supplement: Supplementary file 1 [file viruses-16-00653-s001.zip › Table S3.pdf]

Table S3. Epidemiological data for wild turtles captured in the Bellinger River as a single assemblage, November 2015–November 2020 – univariate analysis and multivariate logistic regression model

		**Inferential test (X^2, Fisher's exact test or Mann Whitney test)**			**Univariate Logistic Model**			**Multivariate Logistic Model**		
**Factors**	**Categories**	**Negative**	**Positive (Prevalence %)**	***p***	**OR (95% CI)**	***p***	**AIC**	**Corrected OR (95% CI)**	***p***	***AIC***
Species	*Emydura macquarii*	310	3 (0.96%)	**<0.001**^			159.88			139.1
	Myuchelys georgesi	164	19 (10.38%)		11.97 (4.01-51.48)	**<0.001**		62.60 (12.93-410.06)	**<0.001**	
Sex	Unknown	225	10 (4.26%)	0.469^						
	Female	127	4 (3.05%)		0.71 (0.19-2.17)	0.567	184.60	Excluded		
	Male	122	8 (6.15%)		1.48 (0.55-3.84)	0.425				
Size	SCL	474	22 (4.44%)	0.469#			183.71			
					1.00 (0.99-1.01)	0.538		1.03 (1.01-1.04)	**0.001**	
Location	Bellingen	279	11 (3.79%)	**0.001***						
	Thora	98	11 (10.09%)		2.85 (1.18-6.85)	**0.018**	170.87	0.87 (0.28-2.51)	0.801	
	Darkwood	97	0 (0%)		0.00	0.988		0.00	0.991	
Year	2015	68	10 (12.82%)	**0.016***			176.83			
	2016	193	8 (3.98%)		0.28 (0.10-0.74)	**0.010**		0.30 (0.09-0.94)	**0.036**	
	2017	100	3 (2.91%)		0.20 (0.04-0.70)	**0.019**		0.18 (0.03-0.74)	**0.026**	
	2018	77	1 (1.28%)		0.09 (0.00-0.48)	**0.022**		0.03 (0.0-0.22)	**0.004**	
	2019	34	0 (0%)		0.00	0.988		0.00	0.994	
	2020	2	0 (0%)		0.00	0.997		0.00	0.999	

A subset (n=496) of the 507 records were included for univariate and multivariate analysis. Statistically significant = **BOLD**, * Fisher's Exact Test, ^ Chi-Square Test, #Mann-Whitney test.
